# Risk assessment of sheep welfare at small-scale slaughter in Nordic countries, comparing with large-scale slaughter

**DOI:** 10.1186/s13028-016-0217-4

**Published:** 2016-06-01

**Authors:** Jan Hultgren, Bo Algers, Sophie Atkinson, Kristian Ellingsen, Sofia Eriksson, Kjartan Hreinsson, Lotta Nordensten, Heidi Valtari, Cecilie Marie Mejdell

**Affiliations:** 1 Department of Animal Environment and Health, Swedish University of Agricultural Sciences, P. O. Box 234, 53223 Skara, Sweden; 2 Department of Animal Environment and Health, Swedish University of Agricultural Sciences, P. O. Box 234, 53223 Skara, Sweden; 3 Department of Animal Environment and Health, Swedish University of Agricultural Sciences, P. O. Box 7068, 75007 Uppsala, Sweden; 4 Veterinary Public Health, Norwegian Veterinary Institute, P. O. Box 750, Sentrum, 0106 Oslo, Norway; 5 Vallnäsvägen 3, 70358 Örebro, Sweden; 6 Icelandic Food and Veterinary Authority, Austurvegur 64, 800 Selfoss, Iceland; 7 Swedish Board of Agriculture, 551 82 Jönköping, Sweden; 8 Brahea Centre for Training and Development, University of Turku, 20014 Turku, Finland

**Keywords:** Abattoir, Animal welfare, Expert opinion, Hazard, Stress

## Abstract

**Background:**

During the pre-slaughter period, animals experience novel environment and procedures which may cause reduced welfare and suffering. Over the last decades, the slaughter industry has restructured into fewer and larger abattoirs, implying potential risks of transport stress, injuries, and impaired animal welfare. Since recently, however, there is growing interest in small-scale slaughter to supply locally or regionally produced meat. Risk managers at all levels thus need to assess animal welfare risks also at small-scale operations. This study aimed to assess risks of poor animal welfare at small-scale lamb slaughter (≤5000 sheep/year and ≤70 sheep/day) in Norway, Iceland, Sweden and Finland, and to compare these risks to large-scale industrial slaughter. Assessment was done applying an individual expert opinion approach during a 2-day workshop. Nine experts in lamb slaughter procedures, behaviour, physiology, health, scoring schemes and/or risk assessment provided estimates of exposure, likelihood of negative consequences following exposure, and intensity and duration of negative consequences for 71 hazards. The methods applied mainly adhered to the risk assessment guidelines of the European Food Safety Authority. The list of hazards was modified from an earlier study and distributed to the experts before the assessment. No other literature was reviewed specifically for the purpose of the assessment.

**Results:**

The highest risks to animal welfare identified in both small- and large-scale slaughter were related to inadequate conditions during overnight lairage at the slaughter plant. For most hazards, risk estimates were lower in small-scale slaughter. The reverse was true for splitting of groups and separation of one sheep from the group.

**Conclusions:**

Small-scale slaughter has a potential for improved sheep welfare in comparison with large-scale industrial slaughter. Keeping the animals overnight at the slaughterhouse and prolonged fasting before slaughter should be avoided. Solutions include continuing education and training of stockpersons and, especially in large-scale slaughter, application of existing techniques for efficient transport logistics that minimise stress.

**Electronic supplementary material:**

The online version of this article (doi:10.1186/s13028-016-0217-4) contains supplementary material, which is available to authorized users.

## Background

During the pre-slaughter period, animals experience novel environment and procedures which may cause reduced welfare and suffering. Since several decades the slaughter industry in many countries has restructured into fewer and larger plants, resulting in longer transports of live animals. This implies an increased risk of impaired animal welfare, as well as meat quality, in sheep and other farm animals, due to stress and injuries [1]. Small-scale slaughter (SS) and mobile on-farm slaughter have attracted interest as means of supplying locally produced meat to consumers [2]. In Sweden small-scale slaughter has increased during the last decade, and 28 new SS plants were approved between 2006 and 2010 [3], which can partly be explained by subsidies to reduce fees for veterinary inspections.

In 2013, 2.1 million sheep were slaughtered in the studied countries, of which 57 % in Norway, 29 % in Iceland, 12 % in Sweden, and 2.4 % in Finland [4]. There were five SS plants (here defined as ≤5000 sheep/year and ≤70 sheep/day), including one mobile unit, and 21 large-scale slaughter (LS, larger than SS) plants for sheep in Norway in 2014 [5]. In Iceland, the first SS plant for sheep was established in 2014. In Sweden, 75 out of totally 82 sheep slaughter plants processed less than 5000 animals in 2014 [6].

Despite positive public expectations, SS may present challenges to both animal welfare and meat quality due to high per-animal costs for investments in appropriate handling facilities, equipment and labour, dependence on the skills of a few stockpersons, lack of established work routines, and limited capacity for e.g. fresh water and cooling in mobile slaughter. There is a need for decision-makers at all levels, from abattoir managers to the European Commission, to assess whether such risks in SS and mobile slaughter differ from those identified in industrial LS. Research is needed to allow producer organisations and the food industry to develop quality assurance programmes and for competent authorities to develop and approve official animal welfare control schemes.

Risk assessment is a set of rational and probability-based methods, applicable to a variety of situations. Guidelines for risk assessment applied to food safety and the control of contagious disease have been presented by the Codex Alimentarius and the World Organisation for Animal Health (OIE). The term “hazard” usually denotes an infectious agent or risk factor potentially causing aversive effects, while “risk” is a function of the probability of occurrence of a hazard and its consequences in case of occurrence. The Animal Health and Animal Welfare Panel of the European Food Safety Authority (EFSA) has issued guidelines for risk assessment of animal welfare [7].

In a graduation project in Veterinary Medicine, Eriksson [8] made a pilot risk assessment of sheep welfare in SS, identifying and characterising important hazards, and collecting preliminary exposure data from seven Swedish SS abattoirs and one Norwegian mobile slaughter plant. Skog Eriksen et al. [9] compared a mobile slaughterhouse with a conventional stationary plant and found animals in the mobile facility to display fewer physiological and behavioural signs of stress. The present work aimed to assess risks of poor animal welfare at SS of lambs in Norway, Iceland, Sweden and Finland, and to compare these risks to industrial LS, using risk assessment and expert opinion.

## Methods

### Data collection

Twenty-two experts (including scientific researchers with varying experience in industry consultancy [n = 15], administrative officials of animal welfare, food control or academic institutions [n = 3], industry consultants [n = 1], quality assurance auditors [n = 1], clinical veterinary practitioners [n = 1], and slaughter plant managers [n = 1]) were invited to a 2-day workshop in Sweden 2012 to estimate risks of poor animal welfare at sheep slaughter. They were identified as knowledgeable and experienced in four areas of expertise; sheep slaughter procedures in general; behavioural, physiological and health-related responses in sheep; scoring schemes in general; or risk assessment in general. In connection with the invitation the experts were asked to fill out and send in a self-evaluation sheet, scoring their own level of knowledge or experience in each of the four areas of expertise as 4 = ”very high”, 3 = ”relatively high”, 2 = ”relatively low” or 1 = ”very low”. Seventeen experts completed the self-evaluation and nine of them (all authors of this paper) agreed to participate in the workshop, including scientific researchers (n = 5), administrative officials of animal welfare, food control or academic institutions (n = 3), and clinical veterinary practitioners (n = 1). All participating experts were known to have practical experience in lamb slaughter, and at least three of them had extensive experience in small-scale slaughter.

The risk assessment was based on individual elicitation of expert opinion in a theoretical exercise. The methods mainly adhered to the guidelines presented by EFSA [7]. The list of hazards was modified from the report by Eriksson [8] and distributed to the workshop participants before the assessment. Detailed instructions were given at the beginning of the workshop. The experts were asked to make all initial assessments individually, i.e. without consulting the other workshop participants, based on their personal experiences and views. Totally 71 hazards were assigned to seven different steps of the slaughter process: unloading and driving to lairage; lairage; driving from lairage to stunning; waiting in stunning box; stunning with captive bolt; stunning with electricity; and bleeding (Additional file 1). Examples of hazards were, (1) during unloading and driving to lairage: stressful handling, long distance, and inappropriate flooring, (2) during lairage: mixing of groups, insufficient bedding, and overnight lairage, (3) during driving from lairage to stunning: stressful handling, inappropriate light, and separation from group, (4) during waiting in stunning box: single sheep, restraint, and spare weapon being kept unloaded in other room, (5) during stunning with captive bolt: poor gun maintenance, incorrectly placed shot, and no restraint, (6) during stunning with electricity: insufficient tong contact, prolonged tong application, and no restraint, and (7) during bleeding: long stun-to-stick interval and insufficient sticking. Animals were assumed to be stunned by either a penetrating captive bolt or electricity. The same hazard list was used in SS and LS. SS was defined as farm-based or handicraft-type slaughter of 5000 or less sheep per plant each year, with a maximum line speed of 70 animals per day. In contrast, LS denoted industrial-type slaughter with a larger number of sheep per year or higher maximum line speed than SS.

Apart from the report by Eriksson [8], no literature was reviewed specifically for the purpose of risk characterisation; it was assumed that the experts had sufficient knowledge of the exposure to different hazards and their effects on animals being slaughtered, and all data were thus provided by the experts during the workshop. No data were collected from slaughter plants. For each hazard and for SS and LS separately, each expert estimated hazard exposure, as well as intensity, duration and likelihood of negative effects given exposure to the hazard, and uncertainty of negative effects based on scientific literature. Hazard exposure expressed the probability of a sheep being exposed to the hazard during slaughter. Likelihood was the probability that a sheep, when exposed to the hazard, would experience the negative effects associated with it. Exposure and likelihood were expressed by three percentages each: the lowest possible (best-case), the most-likely and the highest possible (worst-case) probability. The intensity of the negative effects associated with the hazard was scored as 1 = “negligible”: no or almost no pain, malaise, frustration, fear or anxiety, i.e. the animal behaving normally and quietly, 2 = “mild”: minor pain, etc., some physiological changes, and moderate behavioural changes, e.g. commotion, increased movement or attentive postures, 3 = “moderate”: some pain, etc., some change in motor behaviour, e.g. occasional attempts to escape, and occasional vocalisation, 4 = “severe”: explicit pain, etc., dramatic change in motor behaviour, e.g. violent jumping, attempts to escape or resistance to being restrained, and vocalisation, or 5 = “critical”: fatal effect, with death occurring either immediately or after some time. Duration of the negative effects was scored as 1 (<1 min), 2 (1–5 min), 3 (5–30 min), 4 (30–120 min), or 5 (≥120 min). It was assumed that this period could extend into one or several of the following steps of the slaughter process, up to the point of death.

Expert uncertainty was represented by the range between highest and lowest exposure and likelihood values. Bibliographic uncertainty was scored by the experts (based on their knowledge about available literature) as 1 = “negligible”: solid and complete data available, with strong evidence in multiple references with most authors coming to same conclusions, or considerable and consistent field experience, 2 = “limited”: some or only incomplete data available, with evidence provided in small number of references, authors’ or experts’ conclusions vary, or limited evidence from field observations, or solid and complete applicable data from other species, or 3 = “moderate”: scarce or no data available, with evidence provided in unpublished reports, or few observations and personal communications, and/or authors’ or experts’ conclusions vary considerably.

All in all, the experts were thus asked to complete 1278 spreadsheet entries (two slaughter systems, each with 71 hazards and nine quantities for each hazard). The experts made the scoring on their own laptops using an Excel (Microsoft Corp., Redmond, WA, USA) spreadsheet which was distributed during the workshop. When needed, the procedure was clarified by the principal investigator (first author).

At the end of the first workshop day, all initial estimates were collected and combined by the principal investigator. Medians among the experts were calculated for all quantities. Medians with a standard deviation among expert scores above 0.3 (for exposure and likelihood) or 1.3 (for intensity and duration) were identified as uncertain due to disagreement between experts. On the second workshop day, all values and medians were presented anonymously to the expert group, i.e. without revealing the data source. The exact meaning and implications of hazards and estimates were discussed once more, with emphasis on hazards where there were particularly large differences in interpretation among experts, thus reducing misinterpretation. After the workshop, the experts were allowed to revise and adjust their personal scores for estimates where disagreement or errors had been found. The adjusted scoring sheets were collected by e-mail up to 48 days after the workshop. Logical errors, resulting from adjusting either the lowest, most-likely or highest value of exposure or likelihood without being able to make the necessary adjustments to other values, were corrected by adjusting the original value(s) to maintain the logical order from the lowest to the highest value. For example, if adjustment resulted in a lowest, most-likely and highest exposure of 25, 65 and 50 % after adjusting the most-likely value, the highest exposure was changed to 65 %. Based on the adjusted estimates, new medians were calculated for the final data analysis.

Finally, the experts were asked to describe the slaughter practices in their home countries to the best of their knowledge.

### Statistical analysis

Based on the medians of all nine experts, aggregated magnitude (unitless) was calculated for each hazard as: (intensity × duration − 1)/24. Similarly, aggregated risk (unitless) was calculated as: magnitude × exposure × likelihood/10,000. In this way, both magnitude and risk ranged, theoretically, from 0 to 1. The lowest possible, most-likely and highest possible risks were calculated separately, assuming exposure and likelihood to be independent. A PERT probability distribution was fitted to describe the aggregated risk associated with each hazard. From these distributions, the ranges (difference between highest and lowest) were calculated and the interquartile ranges (IQR; Q3–Q1) were estimated by Latin hypercube simulation in the @Risk 5.5 add-into Excel (Palisade Corp., Ithaca, NY, USA) with 100,000 iterations. The ranges were interpreted as aggregate measures of personal uncertainty regarding the risk. Aggregated magnitude, most-likely risk, risk IQR and risk range were used to make comparisons between hazards, different steps in the slaughter process and slaughter systems; estimates were summarised as minimum, median and maximum values among the hazards included in each step.

The relationship between aggregated magnitude and most-likely risk was investigated by Spearman rank correlation in JMP 9 (SAS Institute Inc., Cary, NC, USA) in SS and LS. Likewise, the relationship between SS and LS was investigated by Spearman rank correlation for aggregated magnitude and most-likely risk. The significance level was set to 0.05.

To investigate how much of the variation in different quantities resided at expert level in different slaughter systems, i.e. how much was due to differences between experts rather than between hazards within expert, eighteen two-level linear mixed models were constructed in JMP, one for each quantity and slaughter system. In all models, hazard was included as the only fixed effect and expert identity as a random effect.

## Results

### Self-evaluation and risk assessment

The score sum of the self-evaluation concerning the four expertise areas ranged among the participants from 6 to 15 (median 12.5). The expert with the highest sum reported a very high level of knowledge or experience in three of the expertise areas (slaughter procedures, scoring schemes, and risk assessment), while the expert with the lowest sum reported a very low score in two areas (behaviour-physiology-health, and scoring schemes). None of the participants reported a very low score for expertise in lamb slaughter procedures in general. For remaining expertise areas, scores ranged from very low to very high.

At the end of the first workshop day, the experts had completed 633 entries (99 %) for SS, and 588 entries (92 %) for LS, as well as at least 87 and 62 %, respectively, of the 71 hazard entries for any quantity. All experts had completed all entries of 60 hazards (84 %) in SS and 23 hazards (32 %) in LS. Hence there were enough data to calculate medians for all hazards and quantities in both slaughter systems. In no case the number of experts that contributed to a median was less than seven. However, a bibliographic uncertainty score of 3 invalidated the aggregated risk estimate for *large group driving* from lairage to stunning (Additional file 1) in LS. When the expert estimates were aggregated, 24 % (SS) and 23 % (LS) of all 568 medians (excluding bibliographic uncertainty) were considered to be uncertain due to disagreement between experts.

After the workshop, 51 entries (9.0 %) in SS and 44 entries (7.8 %) in LS were adjusted by the participants. The overall percentage of entries adjusted varied among the experts between 0.6 and 13 %. The hazards for which entries were most commonly adjusted in SS were *restraint by shoulders and head* during stunning with captive bolt (30 %) and s*pare weapon kept unloaded in same room* during waiting in stunning box (28 %) (Additional file 1). In LS, the hazards with most adjustments were *prolonged tong application* during stunning using electricity (36 %), *no restraint at electrical stunning* (34 %) and *manipulation before sticking*, i.e. animal chained and hung, or moved and placed horizontally before sticking (33 %). Adjustments were made both upwards and downwards; the mean change was 0.001 score points in SS and 0.009 score points in LS for intensity and duration, and 0.8 and 1.1 percentage units for other estimates, respectively. Between 0 and 30 (mean 5.2) illogical probability values per expert were corrected. After all adjustments, the percentage of uncertainty due to disagreement between experts was reduced to 9.7 % of median values in SS and 11 % in LS. Particularly large disagreement was found for three groups of values. First, most-likely likelihood estimates varied between 0–5 and 90–100 % for *large group driving* to stunning, *repeated* *re*-*shot* and *insufficient sticking* in SS. Second, most-likely exposure values varied between 10 and 100 % for *manipulation before sticking* in LS. Third, most-likely likelihood values varied between 0–10 and 95–100 % for *large group unloading*, *large group driving* to stunning, *single sheep* while waiting for stunning, *restraint by wool* while waiting, *spare weapon kept unloaded in other room*, *spare weapon kept unloaded in same room*, *single re*-*shot*, *repeated re*-*shot*, *restraint by neck only* during stunning with captive bolt, *restraint by shoulders only* during electrical stunning, and *insufficient sticking* in LS. Aggregated estimates of magnitudes and most-likely risks after adjustments are shown in Additional file 1.

Table 1 gives descriptive statistics of aggregated magnitude and risk in different slaughter process steps. There were considerable differences between the steps. Magnitude was highest during lairage and lowest during stunning with electricity. Most-likely risk was highest during lairage and lowest during bleeding in SS or during stunning with captive bolt in LS.

**Table 1:** Summary of welfare magnitudes and risks in different steps of slaughter

Process step	Measure	Small-scale			Large-scale		
		Min.	Median	Max.	Min.	Median	Max.
Unloading and driving to lairage (13 hazards)	Magnitude	0.042	0.13	0.33	0.042	0.13	0.27
	ML^a^ risk	0.0042	0.0075	0.03	0.0056	0.023	0.078
	Risk, IQR^b^	0.0025	0.0058	0.018	0.0036	0.013	0.041
	Risk range^c^	0.009	0.021	0.07	0.013	0.047	0.15
Lairage (14 hazards)	Magnitude	0.083	0.33	0.58	0.083	0.33	0.69
	Risk, ML	0.013	0.027	0.13	0.010	0.048	0.18
	Risk, IQR	0.0087	0.018	0.057	0.0097	0.030	0.078
	Risk, range	0.033	0.064	0.20	0.037	0.11	0.32
Driving from lairage to stunning (14 hazards)	Magnitude	0	0.13	0.21	0.042	0.13	0.21
	Risk, ML	0	0.016	0.065	0.0075	0.021	0.06
	Risk, IQR	0	0.011	0.021	0.0057	0.012	0.025
	Risk, range	0	0.038	0.078	0.021	0.045	0.089
Waiting in stun box (10 hazards)	Magnitude	0.083	0.13	0.29	0.083	0.21	0.29
	Risk, ML	0.0044	0.010	0.053	0.0026	0.014	0.083
	Risk, IQR	0.0022	0.0070	0.023	0.0023	0.011	0.036
	Risk, range	0.0079	0.026	0.083	0.0088	0.039	0.13
Stunning with captive bolt (9 hazards)	Magnitude	0.042	0.13	0.29	0.083	0.13	0.29
	Risk, ML	0	0.005	0.038	0	0.0005	0.0095
	Risk, IQR	0.0001	0.0033	0.020	0.0002	0.0008	0.0072
	Risk, range	0.0007	0.012	0.071	0.0013	0.0031	0.026
Stunning with electricity (8 hazards)	Magnitude	0.042	0.10	0.13	0.083	0.094	0.13
	Risk, ML	0.0006	0.0032	0.007	0.005	0.012	0.028
	Risk, IQR	0.0002	0.0019	0.0049	0.0024	0.0063	0.015
	Risk, range	0.0008	0.0078	0.018	0.0088	0.023	0.054
Bleeding (3 hazards)	Magnitude	0	0.13	0.125	0	0.13	0.13
	Risk, ML	0	0.0025	0.005	0	0.0048	0.015
	Risk, IQR	0	0.0024	0.0045	0	0.0033	0.0092
	Risk, range	0	0.0093	0.017	0	0.012	0.033
Totally (71 hazards)	Magnitude	0	0.13	0.58	0	0.13	0.69
	Risk, ML	0	0.010	0.13	0	0.018	0.18
	Risk, IQR	0	0.0072	0.057	0	0.012	0.078
	Risk, range	0	0.027	0.20	0	0.044	0.32

Statistics of estimated magnitudes and risks of poor sheep welfare at small-scale and large-scale slaughter in Norway, Iceland, Sweden and Finland. Hazards arranged in seven process steps, specifying minimum, median and maximum values by process step. Aggregated data from nine experts 2012

^a^Most-likely

^b^Inter-quartile range

^c^Maximum–minimum

**Fig. 1:**
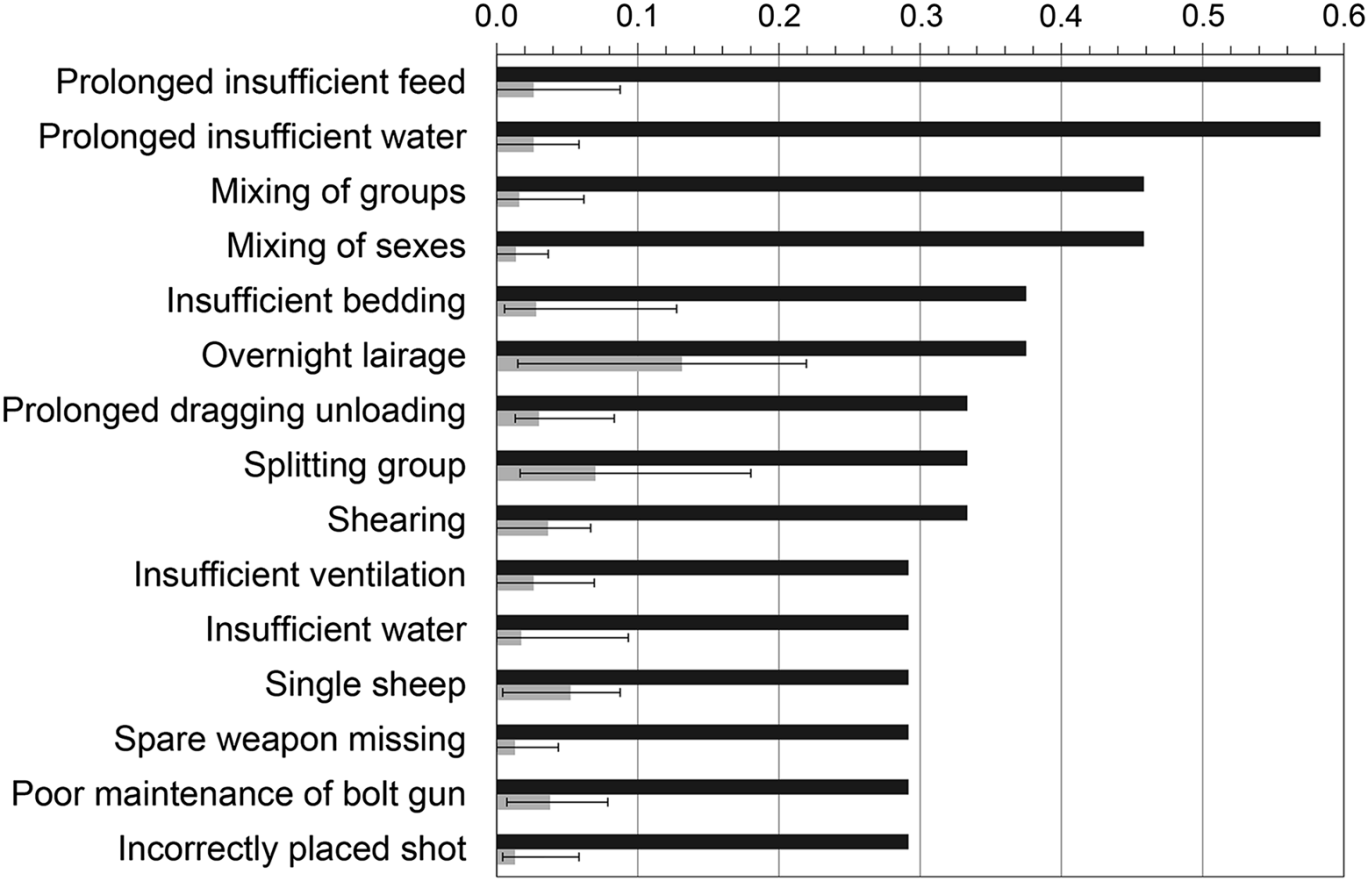
Hazards ranked by welfare magnitude at small-scale slaughter. Sheep welfare hazards at small-scale slaughter in Norway, Iceland, Sweden and Finland; the 15 highest magnitude estimates (sorted by magnitude; *black bars*) and most-likely risk estimates (*grey bars*; *error bars* indicating risk range); aggregated data from nine experts 2012

**Fig. 2:**
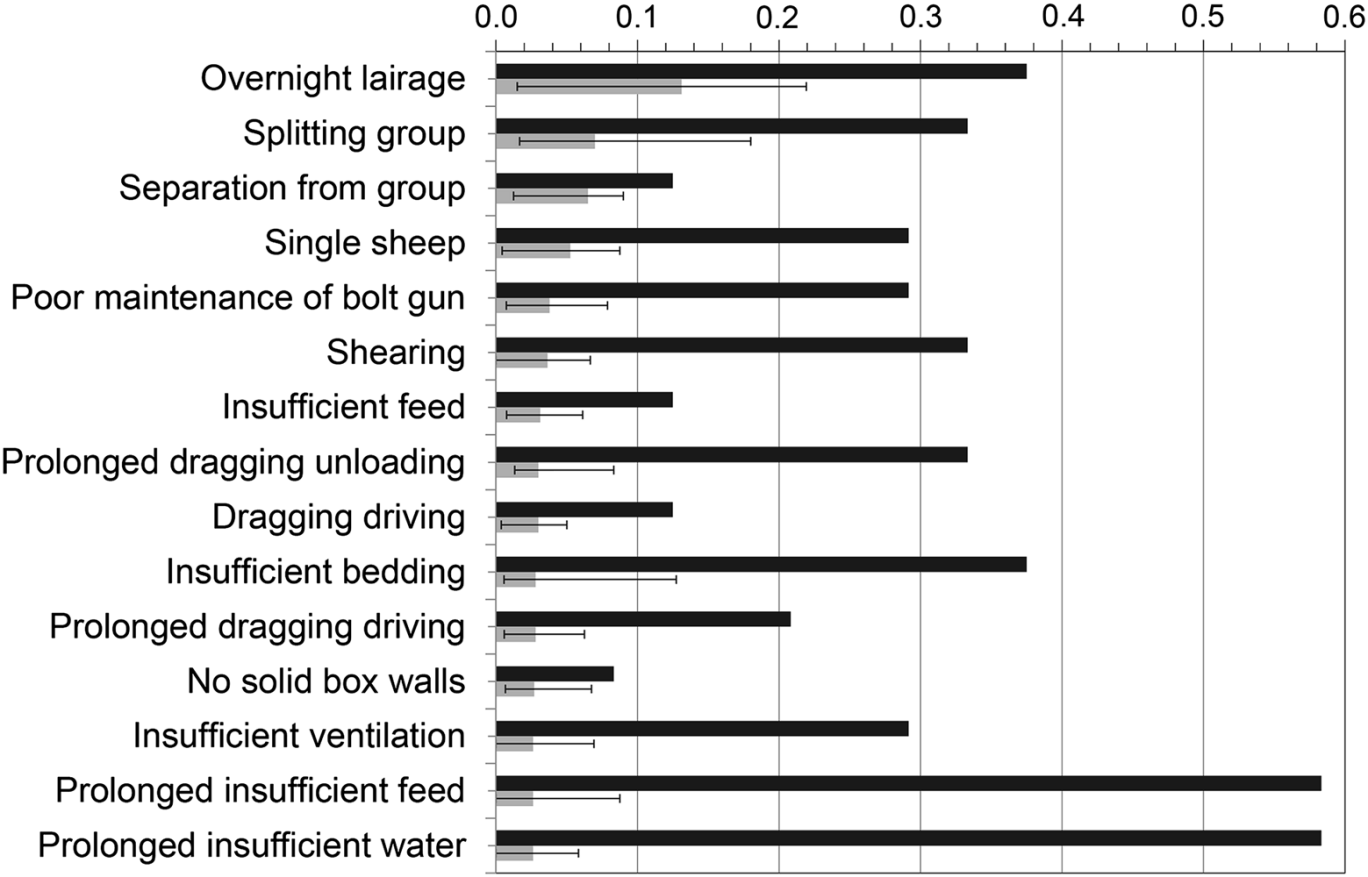
Hazards ranked by welfare risk at small-scale slaughter. Sheep welfare hazards at small-scale slaughter in Norway, Iceland, Sweden and Finland; the 15 highest most-likely risk estimates (sorted by risk; *grey bars*; *error bars* indicating risk range), and magnitude estimates (*black bars*); aggregated data from nine experts 2012

**Fig. 3:**
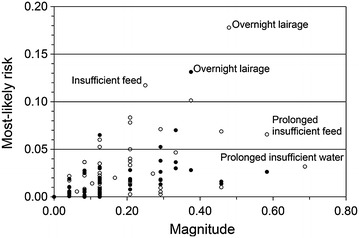
Welfare magnitudes and risks. Relationship between estimates of magnitude and most-likely risk of poor sheep welfare for different sheep welfare hazards at small-scale (*black dots*) and large-scale slaughter (*white dots*) in Norway, Iceland, Sweden and Finland; aggregated data from nine experts 2012

Figures 1 and 2 show hazards with particularly high aggregated magnitudes and most-likely risks in SS. *Prolonged insufficient feed* and *prolonged insufficient water* (Additional file 1) had the highest magnitudes (both 0.58). *Overnight lairage* and *splitting of group* during lairage and *separation from group* during driving to stunning had the highest risks (0.13, 0.070 and 0.065, respectively). In LS, *prolonged insufficient feed* and *prolonged insufficient water* had the highest magnitudes (0.58 and 0.69, respectively) and *overnight lairage* had the highest risk (0.18) (Fig. 3). There was a strong to moderate correlation between aggregated magnitude and most-likely risk in both SS (Spearman *rho* = 0.65; *P* < 0.0001) and LS (*rho* = 0.43; *P* = 0.0002).

**Fig. 4:**
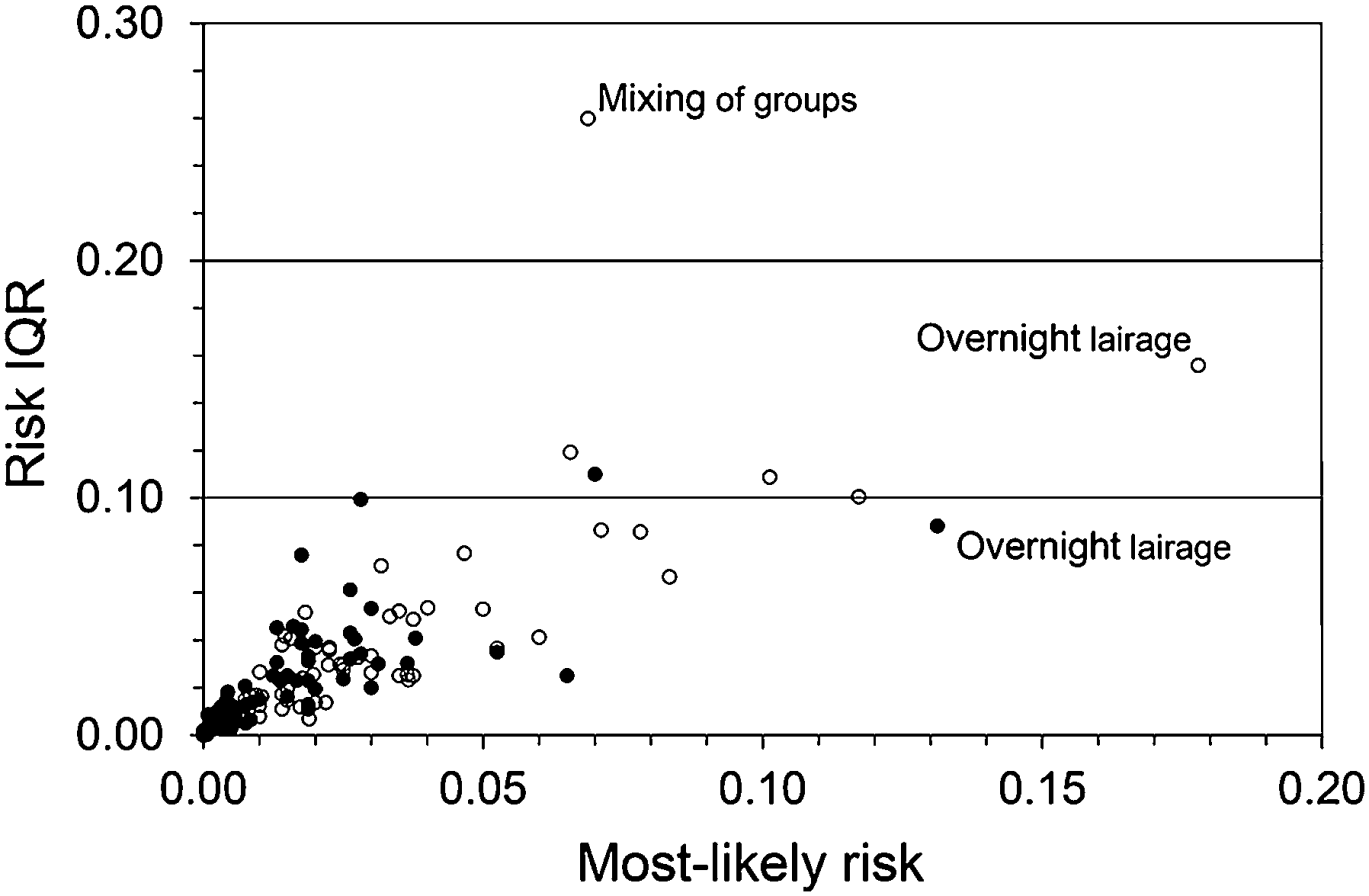
Welfare risks and their interquartile ranges. Relationship between estimates of most-likely risk and interquartile range (IQR) of risk probability distributions of poor sheep welfare (IQR indicating aggregated personal uncertainty regarding the risk) for different sheep welfare hazards at small-scale (*black dots*) and large-scale slaughter (*white dots*) in Norway, Iceland, Sweden and Finland; aggregated data from nine experts 2012

The magnitudes of *overnight lairage* in SS or LS and *insufficient feed* in LS were only moderate but due to high exposure the corresponding estimated risks were high (Fig. 3). The opposite was true for prolonged insufficient feed and water in LS, with high magnitudes but moderate risks. Overall differences in most-likely risk estimates between SS and LS and between slaughter process steps were generally accompanied by corresponding differences in personal uncertainty, as expressed by risk IQR and risk range. Risk IQR (Fig. 4) and range (not shown in figure) were larger when the estimated risk was high. However, there was no obvious association between scores for bibliographic uncertainty and personal uncertainty regarding risks as expressed by IQR of risk distributions.

**Fig. 5:**
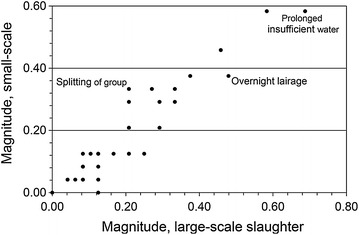
Welfare magnitudes at large-scale and small-scale slaughter. Relationship between magnitude estimates for different sheep welfare hazards at small-scale and large-scale slaughter in Norway, Iceland, Sweden and Finland; aggregated data from nine experts 2012

**Fig. 6:**
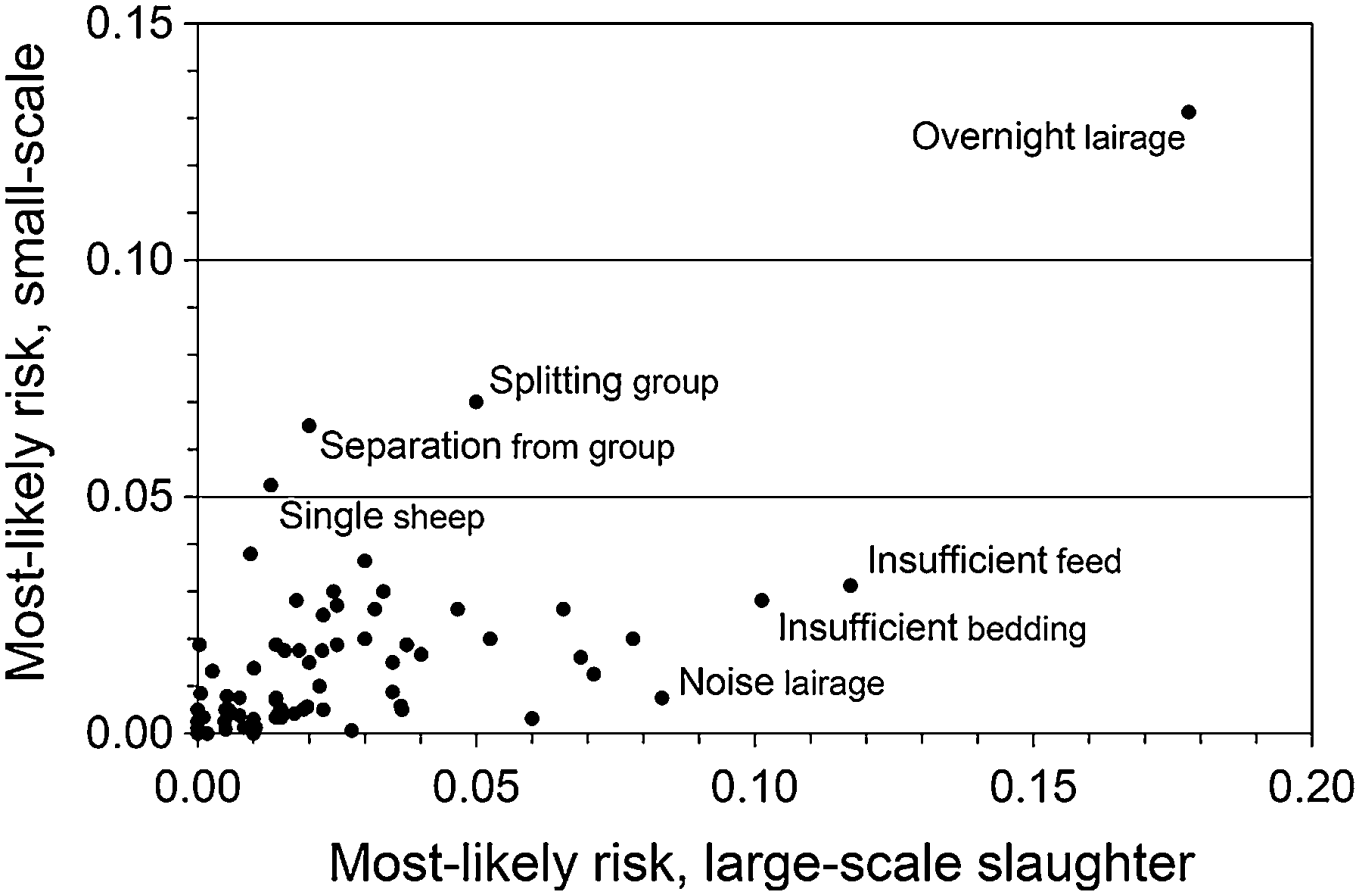
Welfare risks at large-scale and small-scale slaughter. Relationship between most-likely risk estimates for different hazards to sheep welfare at small-scale and large-scale slaughter in Norway, Iceland, Sweden and Finland; aggregated data from nine experts 2012

There were strong to moderate correlations between SS and LS for magnitude (Spearman *rho* = 0.90; *P* < 0.0001) and most-likely risk (*rho* = 0.55; *P* < 0.0001). On average, magnitudes were 8 % lower and risks were 40 % lower in SS than in LS. When comparing SS to LS, *splitting of group* during lairage was associated with a higher magnitude in SS, while *overnight* *lairage* and *prolonged insufficient water* had lower magnitudes (Fig. 5). Most-likely risks were higher in SS for *splitting group*, *separation from group* during driving to stunning and *single sheep* while waiting in stunning box, but lower for *insufficient bedding* and *insufficient feed* during lairage and *noise waiting* in stunning box (Fig. 6).

Estimates from the linear mixed models of expert values are presented in Table 2. Coefficients of variation were similar for SS and LS, and the highest values were found for intensity and duration. For all parameters, variance components for experts were larger in SS than in LS (1.04–7.86 times larger). The highest percentage was found for uncertainty in SS and the lowest for most-likely exposure in LS.

**Table 2:** Summary of linear mixed models of welfare quantities

Quantity	Small-scale		Large-scale	
	R^2^	Expert variance (% of total)	R^2^	Expert variance (% of total)
Lowest possible exposure	0.39	0.0031 (15)	0.43	0.0042 (11)
Most-likely exposure	0.47	0.0039 (11)	0.48	0.00063 (1.4)
Highest possible exposure	0.54	0.012 (21)	0.53	0.0040 (6.3)
Intensity	0.64	0.21 (29)	0.59	0.16 (22)
Duration	0.59	0.12 (15)	0.62	0.034 (5.0)
Lowest possible likelihood	0.48	0.037 (38)	0.45	0.030 (35)
Most-likely likelihood	0.40	0.024 (28)	0.37	0.022 (27)
Highest possible likelihood	0.37	0.017 (25)	0.32	0.016 (22)
Bibliographic uncertainty	0.53	0.22 (46)	0.37	0.14 (30)

Coefficients of determination (R^2^) and expert variance components from linear mixed models of quantities related to sheep welfare risks at small-scale and large-scale slaughter in Norway, Iceland, Sweden and Finland; aggregated data from nine experts 2012

### Slaughter practices in different countries

The participating experts estimated that less than 10 % of the sheep slaughtered in the studied countries were processed at SS, with the highest percentages in Sweden and Finland. For Norway, there were three SS plants in service, including one mobile unit, that electrical head-only stunning was most commonly used (captive bolt in the mobile plant; electricity head-to-back in a few plants), and that overnight lairage was practised frequently. In Iceland, there were no SS or mobile plants and electrical head-only stunning was used primarily. In Sweden, it was estimated that there were around 55 SS plants but no mobile plant. Captive bolt stunning was used in approximately 80 % of the SS plants and head-only electrical stunning in the remaining 20 %. Electrical head-only stunning was used in all LS plants. In Finland, there were around 59 SS plants but no mobile unit, and both captive bolt and electrical stunning were used. In summary, around 6 % of the sheep slaughtered in Norway, Sweden and Finland were processed at SS plants, and approximately two-thirds of these animals were stunned by captive bolt and the remaining third by electricity, most commonly applied across the head of the animals. At least in Norway, shearing is traditionally done at the abattoir, but increasingly commonly it is done after killing.

## Discussion

There was a strong association between SS and LS for aggregated magnitudes, indicating that the hazards were ranked very similarly in the two slaughter systems, while risk estimates were less strongly associated. The magnitudes were comparable in SS and LS, while the risks were considerably lower in SS, mainly due to lower exposure to the hazards. In both SS and LS, hazards to which the animals are exposed during lairage were associated with the highest magnitude and risk estimates, compared to other steps of the slaughter process.

In both SS and LS, the highest magnitudes were found for prolonged insufficient feeding (>16 h) during lairage, and prolonged insufficient watering (>8 h) during lairage. Research on physiological effects of temporary deprivation of feed and water is diverging. Fisher et al. [10] found sheep in different body conditions to adapt to up to 30 h of feed deprivation by mobilising their energy reserves without any evidence of metabolic depletion such as low blood glucose or high meat pH. Inaccessibility of feed for a short period of time (possibly 12–16 h) thus does not seem to have large physiological implications, although the animals may experience some degree of hunger. There is also some evidence that sheep have an ability to withstand prolonged periods of water restriction. According to Silanikove [11] and Jacob et al. [12], the plasma volume and body water balance can be maintained by drawing water into the circulation from the rumen during the first 2 days of water deprivation. In some countries, it is standard practice to fast sheep for a prolonged period before and after transport to slaughter. The main reason for this seems to be a reduced risk of faecal contamination of the fleece and carcass during transport and dressing. Furthermore, New Zealand’s Occupational Safety and Health Service [13] recommends that weaned lambs and ewes are fasted prior to shearing, leaving them without feed for 12–32 h and without water for 8–24 h, to avoid discomfort, stress, defecation and urination during handling at shearing. Our results to some extent contradict these recommendations, although the discomfort at shearing may be very different from that experienced during handling before slaughter [14].

Other hazards with high magnitudes were mixing of animals from different transports and introduction of an adult ram into the group during lairage. When groups of sheep unfamiliar to each other are mixed, they keep to their own groups for several weeks before full integration occurs [15].

In terms of risk, overnight lairage was associated with the highest estimate in SS, almost twice as high as the second highest estimate, although the magnitude was moderate. Existing research on effects of abattoir lairage time on animal welfare is limited and results are inconsistent. Some studies show that lairage after transport potentially allows animals to decrease the concentrations of stress hormones, restore muscle glycogen concentrations and reduce dehydration [1, 16]. Other studies suggest that lairage conditions themselves may prevent animals from resting and recovering from feed and water restriction [17]. Overnight lairage received a considerably lower risk estimate in SS than in LS, but also a somewhat lower magnitude, possibly indicating that the lairage conditions were considered to be superior in SS.

In Europe and North America, sheep are usually slaughtered on the day of arrival to the abattoir, whereas in e.g. Australia, New Zealand and China sheep are more typically slaughtered the day after arrival [18]. In the Nordic countries, sheep farms are often small and located at some distance from the abattoir. Hence, it may be difficult to collect enough sheep to start LS in the early morning without overnight lairage. However, research has shown that this is usually a result of poor logistics [19]. The application of modern logistics technology, such as specialised software and central coordination of contracted vehicles, has been shown to allow for higher efficiency, better animal welfare and lower emissions in Swedish farm animal transport [20].

Beside overnight lairage, main risks identified in SS included splitting of groups during lairage, separation of one sheep from the group during driving to stunning, and isolating single sheep while waiting for stunning. These hazards were associated with only low to moderate risk estimates in LS. In SS, the groups are smaller than in LS, implying that a larger proportion of sheep will experience being the first animal taken out or the last one left in a group. Sheep react on being isolated from their flock mates by agitation, escape (or immobilisation), vocalisation and a physiological stress response [21, 22]. Baldock and Sibly [23] found that isolation from the flock resulted in a stronger stress response than transportation did. In contrast, Hargreaves and Hutson [14] did not detect changes in heart rate, plasma cortisol or haematocrit as a stress response to 4 min of isolation, and suggested that individual handling and familiarity with the procedure may have attenuated this response. Isolation is less stressful if the sheep are able to see other sheep nearby [24].

In LS, particularly high risk estimates were obtained for insufficient feeding (no feed available for >2 h), insufficient bedding (hard, wet or dirty flooring), and mechanical noise (sound levels of >75 dB from metal fittings or ventilation) during lairage. The importance of bedding quality to maintain welfare depends on whether lairaged sheep are newly sheared; not fully fleeced ewes show a preference for a soft lying surface with low thermal conductivity [25]. Hall et al. [26] found that excess noise during transportation had effects on salivary cortisol, heart rate and behaviour.

Eriksson [8] classified hazards as of “high”, “lower” or “negligible” risks and found overnight lairage to be associated with relatively high risks in comparison with other hazards, which is in agreement with our risk assessment. The author found dragging or pulling of animals by wool, horns, ears or tail for >10 % of the time when driving from lairage to stunning to be the main risk. Algers et al. [27] presented no risk estimates for hazards in sheep slaughter, and did not mention overnight lairage as a hazard. EFSA [28] concluded that research is needed to develop methods for restraining single sheep with minimal stress to the animal prior to stunning and methods to maintain good electrical contact with the stunning equipment, but did not make a complete hazard identification. Neither Algers et al. [27], nor EFSA [28] addressed SS or mobile slaughter specifically.

Clearly, many of the hazards included in this study are related to the competence and skills of the abattoir stockpersons and the slaughter line speed. For example, insufficient feeding and incorrect grouping of animals may be caused by human shortcomings. Likewise, a lower line speed allows stockpersons to care for individual animals and take extraordinary time-consuming measures when required. The amount of distress that sheep suffer during handling is likely to be affected by the quality of the stockperson [29–31], and the time available for appropriate handling of animals [32]. EFSA [33] concluded that an inability to understand animal needs, infrequent inspection to assess whether animal needs are being met, inspections where animals cannot be properly observed or too many animals per stockperson can all lead to poor management decisions that may impact on sheep welfare. In addition, behavioural reactivity to human handling may be affected by previous handling [34] and breed.

Typically, risk assessment produces separate estimates of magnitude and risk for each hazard, with limited possibilities to account for interaction between hazards, as discussed by EFSA [7]. In reality, multiple interactions are usually present. For example, simultaneous exposure to separation from flock mates, stressful handling and high noise levels may produce a larger decrease in welfare than is indicated by the three separate magnitude and risk estimates. According to Hutson and Grandin [31], it is likely that sheep flock more tightly together if they are fearful. Stress may also accumulate during the slaughter process, and stress caused by hazards in early steps may increase susceptibility to hazards appearing later on. For example, animals exposed to overnight lairage, prolonged fasting or rough handling when arriving at the abattoir are probably more vulnerable to welfare hazards in connection with stunning. The present study did not consider interactions between hazards or cumulative effects.

For some hazards, the scientific evidence of exposure and adverse effects on sheep at slaughter is scarce, making risk assessment difficult. Furthermore, the current study relied completely on the expertise and experience of nine experts who contributed with individual estimates of exposure to hazards, and intensity, duration and likelihood of negative effects. Another group of experts may have generated different estimates, due to different levels of knowledge, experience and personal values. It is obvious that pre-conceived personal preferences for SS may have biased the comparison with LS. However, the 22 experts invited to the workshop were among the most knowledgeable persons in the studied countries, and nine of them provided the data.

The results also depend on the way hazards were specified and explained at the workshop, and how they were interpreted and conceived by the participants. It is not unlikely that some of the large disagreements between experts were due to misunderstanding or misconception although, during the workshop, care was taken to discuss different hazards and types of estimates in order to reach a common understanding. The experts were allowed to adjust their estimates for quantities with a high degree of disagreement, which was shown to increase agreement and can be expected to have improved the quality of final estimates. Still, even after these adjustments, major disagreements were found for a number of hazards regarding likelihood values in SS and both exposure and likelihood estimates in LS. Although none of these differences concerned hazards with particularly high magnitude or risk estimates, they show that experts may disagree strongly if they are allowed to express personal opinions. This underlines that selection and elicitation of experts is a delicate task which requires great care to enable reliable results. Furthermore, in consensus assessment, disagreements may probably be hidden by opinions that are expressed forcefully by the most dominant individuals in a group of experts.

The current risk assessment applies to all sheep processed by SS or LS in Norway, Iceland, Sweden and Finland. Estimates of exposure, intensity, duration and likelihood were averaged over all conceivable slaughter plants in the four countries. In practice, the circumstances, and thus the animal welfare risks, may vary heavily between different plants and regions, due to differences in sheep breeds, farming structure, slaughter industry structure, transport conditions, educational standards, legislation and tradition. Norway and Iceland are not part of the EU, in contrast to Sweden and Finland, but are closely connected through similarities in regulations and practices. For the same reasons, the risks identified in this study may differ from other countries where SS of sheep is applied.

## Conclusions

From risk assessment based on expert opinion, we conclude that overnight lairage at the slaughter plant is associated with the strongest negative welfare effects at both small- and large-scale sheep slaughter in Norway, Iceland, Sweden and Finland. We also conclude that prolonged insufficient feeding and watering during lairage potentially are the largest threats to animal welfare, depending on their occurrence. Small-scale slaughter appears to have a potential for better animal welfare than large-scale slaughter. For most factors relevant to welfare, the risks are higher in large-scale than in small-scale slaughter, except for splitting of groups during lairage, separation of one sheep from the group during driving to stunning, and single sheep while waiting for stunning. In small-scale plants, a comparatively low line speed probably allows more consideration for the individual needs of animals being slaughtered.

## Additional file

**Additional file 1. **Aggregated risk assessment data. Process steps, hazards and their estimated magnitudes and most-likely (ML) risks of poor sheep welfare at small-scale and large-scale slaughter in four Nordic countries; aggregated data from nine experts in 2012.

## Abbreviations

EFSA: European Food Safety Authority
IQR: interquartile range
LS: large-scale slaughter
ML: most likely
OIE: World Organisation for Animal Health
SS: small-scale slaughter
